# mSphere of Influence: Host-pathogen interactions—the fascinating world of molecular interplay

**DOI:** 10.1128/msphere.00907-25

**Published:** 2026-04-22

**Authors:** Dilip Kumar

**Affiliations:** 1 Department of Biology, Trivedi School of Biosciences, Ashoka University, Haryana, India; Virginia-Maryland College of Veterinary Medicine, Blacksburg, Virginia, USA

**Keywords:** host-pathogen interactions, RNA virus, structural biology

## Abstract

Dilip Kumar is a structural biologist, working on developing novel antiviral strategies against pathogenic RNA viruses by using an integrated structural biology approach. In this mSphere of Influence article, he emphasizes how two remarkable research articles on host-pathogen interactions, “Inhibition of IRGM establishes a robust antiviral immune state to restrict pathogenic viruses” by P. Nath, N. R. Chauhan, K. K. Jena, A. Datey, et al. (EMBO Rep 22:EMBR202152948, 2021, https://doi.org/10.15252/embr.202152948), and “Cotranslational prolyl hydroxylation is essential for flavivirus biogenesis” by R. Aviner, K. H. Li, J. Frydman, and R. Andino (Nature, 596:558–564, 2021, https://doi.org/10.1038/s41586-021-03851-2), sparked his interest in pursuing host-pathogen interactions to develop a broad range of antiviral therapeutics.

## COMMENTARY

My journey in the biological sciences began with my very first experiment on bacterial transformation in a master’s lab course on molecular biology. The idea of transforming a cell by introducing a foreign plasmid DNA to make it translate your protein of interest was a fascinating concept for me and a reason for me to delve into the field of structural biology.

During my PhD, I worked on the structural and functional characterization of mycobacterial nanoRNase, an enzyme responsible for hydrolyzing small RNA fragments (2–5 mer). I determined the first high-resolution structure of a mycobacterial nanoRNase using the selenomethionine SAD phasing method (1). Later, I moved to Baylor College of Medicine, USA, for my postdoctoral research, where I focused on the gastrointestinal viral pathogen, rotavirus. My work aimed to understand transcription and RNA capping in rotavirus. I determined the first full-length cryo-EM structure of the rotavirus capping machinery, VP3, which contains all the enzymatic domains required for RNA capping (2). I also determined the X-ray structure of rotavirus VP3 and used the cryo-EM model to obtain phase information for the crystallographic data.

My interest in host–pathogen interactions deepened during the coronavirus disease 2019 (COVID-19) pandemic, when I encountered a study by Nath et al. demonstrating that suppression of the IRGM gene renders human cell lines and mice resistant to a range of viral infections. IRGM is a host factor inversely related to interferon expression; depletion of IRGM enhances the levels of key viral restriction factors, such as IFITMs, APOBECs, SAMHD1, tetherin, viperin, and HERC5/6, thereby conferring protection against multiple viral infections, including SARS-CoV-2 and Zika virus (3). The IRGM knockdown in mice provides them immunity against chikungunya virus infection (3). This study highlighted the delicate balance in host–pathogen interactions that can shift outcomes toward either pro- or antiviral effects, opening new avenues for therapeutic intervention.

In another compelling study, by Aviner et al., published in *Nature*, the authors performed polysome profiling and proteomic analyses of virus-infected cells (Poliovirus, Zika virus, and dengue virus). They observed the hydroxylation of conserved proline residues in flaviviruses (Zika and dengue viruses) polyprotein, and inhibition of host proline hydroxylases, specifically ER proline hydroxylases (P4HA1 and P4HA2), suppresses viral growth, suggesting the proviral effect of this modification (4). The proline hydroxylases, ER-resident enzymes, are canonically involved in collagen maturation within the endoplasmic reticulum. During flavivirus infection, these enzymes co-translationally catalyze the post-translational modification of conserved proline residues in viral polyprotein inducting a proviral effect, which is an elegant example of how pathogens exploit host machinery.

Neutralizing antibodies represent another powerful component of the host defense arsenal with immense therapeutic potential against infectious disease. Advances in protein large language models trained on antigen-antibody structures and interactions are now enabling the design of *de novo* single-chain antibodies (5, 6) with the potential to significantly accelerate antiviral development. Together, these studies, in addition to many such recent studies, have opened exciting avenues for antiviral strategies. Furthermore, the developments in mass-spectrometric, cryogenic electron tomography methods and ML-based computational approaches may allow us to revisit the interactome of host-pathogen interactions. These advances will deepen our insights into molecular crosstalk and may guide the development of more effective broad-spectrum antivirals.
